# Cauterization of the root of the left coronary artery as a straightforward, large and reproducible ischemic injury model in neonatal mice

**DOI:** 10.1038/s41684-024-01443-x

**Published:** 2024-10-22

**Authors:** Tianyuan Hu, Bernd K. Fleischmann, Mona Malek Mohammadi

**Affiliations:** Institute of Physiology I, Life and Brain Center, Medical Faculty, University of Bonn, Bonn, Germany

**Keywords:** Cardiac regeneration, Heart failure, Cardiac regeneration, Heart failure

## Abstract

The adult mammalian heart is known to have very limited regenerative capacity, explaining at least in part the frequency of cardiovascular diseases and their impact as the leading cause of death worldwide. By contrast, the neonatal heart has the ability to regenerate upon injury, and the molecular mechanisms underlying this regenerative capacity are intensely investigated to provide novel cues for the repair of the adult heart. However, the existing rodent neonatal injury models—apex resection, left anterior descending artery ligation and cryoinjury—have limitations, such as being technically demanding, yielding a nonphysiological injury type and/or lack of reproducibility. Here we have therefore established a novel ischemic heart injury method in neonatal mice via cauterization of the root of the left coronary artery. This surgical procedure is technically straightforward, requires less than 10 min for completion and yields reproducible, large ischemic lesions (40% of the left ventricle) with low mortality rates (10% of animals). The injury also induces secondary pulmonary hypertension shortly after surgery, allowing to study the response of the right ventricle. Moreover, neonatal mice at postnatal days 1 and 3 display strongly opposing outcomes after the surgery, because of the lack of cardiac regeneration at the later stage. Thus, this new neonatal heart injury model is of great use for mechanistic studies exploring the regeneration of the left ventricle and the adaptation of the right ventricle upon myocardial infarction.

## Abstract (web summary)

This Protocol describes a novel ischemic heart injury method in neonatal mice via cauterization of the root of the left coronary artery. The procedure is technically straightforward, requiring less than 10 min per mouse for completion.

## Introduction

The limited regenerative capacity of the human heart due to low cardiomyocyte (CM) renewal rate, which further deteriorates with age^1^, poses a substantial challenge contributing to the prevalence of cardiovascular diseases (CVDs) as the leading cause of death worldwide. New therapeutic strategies are therefore needed to unlock the regenerative ability of the adult heart and reduce the burden of CVDs. To identify new therapies, researchers have used model organisms with endogenous regenerative ability such as the zebrafish^2^ and, more recently, the neonatal mouse^3^. Studies have shown that neonatal mice at postnatal day (P)1 can regenerate their heart, but this ability is lost 7 days after birth^3^. The regenerative response of P1 mice is characterized by enhanced CM proliferation, angiogenesis and minimal hypertrophy or fibrosis^3^. However, at P7 the lack of regeneration coincides with CM cell-cycle arrest. Unraveling the regenerative mechanisms of the heart, which seem to be part of a complex process involving an interplay between different cell types, as well as transcriptome and epigenetic changes^4,5^, requires further mechanistic studies. For this purpose, a reliable and physiologically relevant injury model is critical. To this aim, different injury models have been developed, each with advantages and disadvantages. While apex resection and cryoinjury are not ischemic lesions, the induction of myocardial infarction (MI) through left anterior descending artery (LAD) ligation can very well mimic the physiological condition of an acute ischemia and an infarct. However, LAD ligation presents challenges regarding its success rate and the reproducibility of injury size due to suture positioning and/or diverse coronary patterning in mice^6^. Given the technical difficulties of the surgery, the method can only be performed by experts. Here, we describe a new MI model in neonatal mice by cauterizing the entire left coronary artery (LCA). This newly established neonatal MI model is easy and fast to perform, is reproducible and generates a large ischemic injury in the myocardium without damaging the epicardial or endocardial layer of the heart. Our protocol describes all the details necessary to successfully perform the surgery and aims to make it accessible to a broader range of scientists interested in cardiovascular regeneration. The ultimate goal is to uncover the mechanisms of cardiovascular regeneration in neonatal mice with the aim of discovering therapeutic strategies for the human heart and reducing the burden of CVDs in the future.

When employing this surgery model, we unveiled the ability of P1 mice to regenerate after such a large ischemic injury, whereas this ability was already impaired in P3 mice^7^. Despite both P1 and P3 mice experiencing the same initial injury size, P1 mice exhibited enhanced CM proliferation rate, angiogenesis and protective mechanisms against global activation of apoptosis, whereas P3 mice showed low degrees of CM proliferation and angiogenesis and enhanced apoptosis, resulting in left ventricular dilation and heart failure at 7 days post surgery (dps). Furthermore, the large ischemic lesion induced secondary pulmonary hypertension as a consequence of the left ventricle (LV) failure, raising a prominent adaptive response in P1 right ventricle (RV) characterized by enhanced CM proliferation and angiogenesis, without deterioration of RV function. In P3, however, the RV showed a maladaptive response characterized by dilation, reduced wall thickness and CM hypertrophy as well as reduced function together with LV failure as early as 1 dps. Thus, cauterization of the LCA serves not only as a LV MI model but also as a model of secondary pulmonary hypertension, which is the most common form of pulmonary hypertension leading to RV failure in adults^7^. Likewise, and regardless of the underlying pathology, persistent pulmonary hypertension is an important clinical problem in newborns^8^.

### Development of the method

Following the discovery of the regenerative ability of the neonatal mouse heart upon apical resection^3^, numerous studies have aimed to explore the underlying mechanisms of heart regeneration by implementing this surgery technique. Although this method seemed easy to perform, reproducibility was challenging due to variations in scar size^9,10^. Consequently, cryoinjury employing a defined cryoprobe was established to standardize the method and injury size^11,12^. However, it soon became evident that this method was not ideal and did not recapitulate the typical features of ischemic lesions. With this method, a necrotic scar is generated that does not fully resolve and persists much longer akin to what has been reported in the zebrafish^11,13,14^. The different sizes of cryoprobes and different durations of cryoprobe application made it possible to investigate the extent of regeneration in the neonatal mouse heart. These studies showed that neonatal mice cannot fully regenerate the heart upon transmural cryoinjury^11,14^. In an effort to generate a more physiological injury resembling an ischemic MI, LAD ligation in P1 mice was established^15^. The technical challenges associated with this method, in particular the need for microsurgery skills, have made it less accessible to the broader scientific community. In addition, concerns persist regarding the reproducibility of the scar size, as it highly depends on the positioning of the suture and the anatomy of the mouse coronary arteries^6,15–17^. Therefore, the success of the surgery and infarction size require careful monitoring via echocardiography.

We aimed to establish an MI model that not only circumvents the technical hurdles of LAD ligation, but also yields larger scars to facilitate the evaluation of the extent of neonatal heart regeneration and underlying mechanisms following ischemic injury. We thought that a large ischemic lesion could have also the advantage of narrowing the regenerative window and thus facilitate the interpretation of the results. We therefore opted for cauterization of the proximal site of the LCA instead of suturing more distally the LAD. Cauterization of a vessel is not a new term or technique; it has first been described in the Edwin Smith Papyrus, an ancient Egypt medical textbook (c. 1600 bc)^18,19^, known as ‘Secret Book of the Physician’. Drawing inspiration from this ancient technique, we employed cauterization to innovate a new injury model.

### Applications of the method

Given the simplicity of the cauterization compared with suturing, we opted to target the main LCA precisely at the root. This approach generates a larger and more reproducible ischemic injury compared with LAD ligation, enabling to study the degree of regenerative capacity in neonatal mice. Moreover, this method proves to be easier to perform compared with LAD ligation, given the thickness and clear visibility of the main coronary artery. It is important to note that the cauterization method is versatile, as it can be also used to generate a smaller ischemic injury based on the location of cauterization. Moreover, it induces an injury across the entire length of the myocardium in the LV, while preserving an intact endocardium and epicardium, enabling the investigation of the role and activation of both the epicardium and endocardium in the regenerative process. For instance, this facilitates the assessment of coronary artery development from the endocardium as suggested before^20,21^. Given that the coronary artery stem is completely occluded, there is hardly any blood supply to the LV, unlike the LAD ligation^22^, thereby providing an opportunity to assess the full capacity of coronary artery regeneration. Moreover, with this method, the induction of a substantial injury size and rapid onset of LV failure following cauterization leads to a rapid onset of secondary pulmonary hypertension. Notably, we have discovered a prominent adaptive response and plasticity of the RV in P1 mice, which was absent in P3 mice^7^. Given that secondary pulmonary hypertension is the predominant form of pulmonary hypertension, this model holds a unique potential to further investigate the adaptive response and plasticity of the RV in P1.

### Comparison with other models

Apex resection as the first model to study cardiac regeneration aims to remove 15% of the LV and only targets the apex area of the LV^3,23^. However, this model is not ischemic, causing neither apoptosis nor necrosis. In response to the apex removal, a blood clot forms sealing the apex of the heart, which is gradually absorbed and replaced with new myocardium^3^. Although intriguing, this model lacks physiological relevance, the mortality rates can be relatively high when the resected area exposes the chamber, and the technique is difficult to standardize, resulting in potential differences in injury sizes among the operated mice.

By contrast, cryoinjury, which is easy and quick to perform, provides a standardized injury size with a clearly demarcated border zone. Despite its nonphysiological nature, cryoinjury results in a rigid necrotic area accompanied by an extensive inflammatory response that persists in the heart. Cryoinjury using a 1.5 mm cryoprobe in P1 mice has been reported to generate a larger injury compared with apex resection and LAD ligation, corresponding to 18% of the myocardium. The injury, which is transmural, thus damages epicardium, myocardium and endocardium of the heart, leading to the failure of P1 mice to regenerate the injury completely^11^. However, it remains unclear whether the lack of regeneration upon large transmural cryoinjury is due to the nature of the injury, with its necrotic scar that is hard to resolve, or to the degree or depth of injury.

As mentioned above, LAD ligation stands out as the optimal model for MI owing to its physiological nature and its ability to induce ischemic cell death in the myocardium. However, the placement of the suture beneath the LAD is technically challenging. Suturing appropriately is critical, as the suture must be positioned not too deep, yet deep enough to be underneath the LAD. In addition, the knot has to be tight enough to ligate the LAD without being overly tight, as excessive tension could lead to the rupture of the delicate cardiac tissue in neonatal mice^17^. In the first LAD ligation protocol described, the LAD was not always visible, and the suture had to be placed without visualization, based on the anatomical location of the LAD^23^. The method was then refined, involving ligation of the LAD above branching, thereby causing minimal damage to the surrounding tissue and resulting in an infarct size ranging from 10% to 15% of the LV^17^. Conversely, a deep suture can lead to a permanent transmural scar at the ligation site, associated with structural changes^15,17^. Therefore, scarring at the suture location, persisting in the heart, complicates the assertion of ‘full regeneration’. It remains unclear whether a scar is the result of the suture remaining in the heart or rather of a limited regenerative capacity. Beyond the challenge of reproducibility of injury size based on suture positioning, the success of LAD ligation and MI is not guaranteed if the suture is too superficial or fails to locate under the LAD owing to poor visualization or insufficient depth. Therefore, it is imperative to perform an echocardiography one day after LAD ligation to confirm LV failure and exclude those animals in which the procedure was not successful.

Cauterization, akin to LAD ligation, generates a nontransmural injury, but the injury is larger than reported before in neonatal mice, namely almost 40% of the LV. This injury model suggests that it is not just lesion size, but rather the loss of the endocardium and epicardium observed with cryoinjury, that might limit the regenerative capacity of the neonatal mouse heart. The cauterization method targets the root of the LCA, which is clearly visible and is the thickest part of the artery. This ensures precise location for the injury as this large vessel, unlike the LAD, does not easily fade away. Thus, this model is characterized by its simplicity, reproducibility and the larger injury size compared with all the other existing injury models. A cauterization approach has been also reported in zebrafish heart^24^; however, it has been used to generate a necrotic area in the heart by burning the tissue rather than inducing ischemia by ablating a coronary artery, owing to the difficulty of visualizing such vessels in zebrafish hearts. Thus, the cauterization approach combines the advantages of the simplicity and reproducibility of cryoinjury with the more physiological nature of the ischemic LAD ligation.

The anesthesia and analgesia regimen align with those utilized in other neonatal surgery models, and endotracheal intubation and mechanical ventilation are not required. In ensuring the survival of neonatal mice postsurgery, aside from successful surgery procedure, the duration of the surgery and hypothermia emerge as critical factors. Hypothermia, which is the main anesthesia method used for neonatal mice before and during surgery, is generally well tolerated by the mice. Nevertheless, prolonged hypothermia can reduce the recovery rate of the pups. Notably, the cauterization method proves to be relatively swift, as it is straightforward to visually identify the root of the LCA and a suturing procedure is not required. This, in turn, can reduce intraoperative mortality due to prolonged hypothermia and can also speed up the recovery of the mice. The surgical survival rate following cauterization (90%) is similar to cryoinjury, but cannibalization can add 10–20% more mortality, as seen with other neonatal injury models^23^. Importantly, we have optimized the procedure to suture the skin invisibly by concealing the suture within the skin layer. This method prevents maternal access or removal and has proven to reduce the cannibalization rate, which is known to be a major detrimental factor in neonatal mouse surgeries.

In most neonatal injury models, P3 is already considered a nonregenerative stage owing to the strong decline in the regenerative ability upon injury^7,25^. Although the injuries are tolerable for P3 and P7 mice, they are associated with a lack of regeneration and persistent scar^3,15^. By contrast, cauterization injury is barely tolerable for P3 mice as most of them die or need to be killed within the first week of surgery. Mechanistically, we observed similar regenerative mechanisms upon cauterization compared with other injury models: LV regeneration at P1 following cauterization seems to involve CM proliferation, angiogenesis and tolerance to stress-induced apoptosis. While these mechanisms were strongly diminished in P3, bulk RNA sequencing of the LV at 1 dps showed a high overlap among the overexpressed genes in P1 and P3 mice. However, the main distinctive feature between P1 and P3 was the response of the RV, not only at the cellular but also at the molecular level. In P1, unlike P3, the RV exhibited an adaptive response, characterized by enhanced CM proliferation and angiogenesis^7^.

### Overview of the procedure

The procedure comprises five major steps: preparation for the surgery including disinfection and application of analgesia (Steps 1–3), surgical procedures (Steps 4–19), postsurgery analgesia and monitoring (Step 20), confirmation of the surgical success by echocardiographic assessment (Steps 21–27) and killing of the mice followed by organ removal and processing of the hearts (Steps 28–38).

### Experimental design

We strongly recommend following the ARRIVE guidelines for reporting animal research to improve transparency, and reproducibility of experiments^26^. It is important to also report mouse strain, mouse maintenance conditions and the approved animal protocol number alongside the scientific results.

In our laboratory, we have extensive experience with neonatal cardiac surgery models such as neonatal model of transverse aortic constriction as well as different MI models (cryoinjury, LAD ligation and cauterization of the root of the LCA)^7,12,27,28^. For the surgery described herein, we used neonatal mice from a mixed ICR/CD1 and C57BL/6 background, because the litter size is bigger than mice with a pure C57BL/6 background. One advantage of our method is that the size of the pups is not relevant, and the method is applicable to pups of different sizes. We also optimized the suturing strategy to reduce cannibalization. In any case, we recommend ICR/CD1 mothers to act as foster mothers whenever possible, because they have a good nursing behavior. We also suggest allocating the entire litter to sham or cauterization surgery to avoid cannibalization of cauterized mice due to different behaviors. Cannibalization is mainly observed within the first 24 h after surgery. We have not observed any difference in the difficulty for performing the surgery or the outcome between male and female mice. For animal welfare reasons, regular (daily, for a week) monitoring of operated mice is needed; in case of severe heart failure, no feeding and/or gain of weight, the pups need to be killed to prevent suffering.

We have monitored cardiac function in the mice with echocardiography at 1, 7, 14, 21 and 120 dps, measuring ejection fraction (EF), fractional shortening (FS), RV fractional area change (RVFAC), RV end diastolic and systolic area (RVEDA and RVESA), LV wall thickness, LV end-diastolic area (LVEDA) and cardiac output (CO) immediately after surgery and during the regeneration process. Postkilling immunostaining and/or Sirius Red staining was performed to evaluate the scar size at 2, 7 and 21 dps and assess other parameters such as CM proliferation or angiogenesis. To assess scar size at early postsurgery time points, such as 2 dps, Sirius Red staining is unsuitable due to insufficient collagen formation. Instead, the scar can be detected by performing TNNI3 (Troponin I) staining and measuring areas lacking TNNI3, or by using terminal deoxynucleotidyl transferase dUTP nick end labeling (TUNEL) staining to identify regions containing dead cells.

### Level of expertise required

When learning the surgery, it would be important to know the anatomy of the heart and, in particular, the anatomic location of the LCA to be able to identify it with a minimal skin muscle incision and manipulation. Thus, practicing in postmortem animals is recommended and would help the learning process. In our hand, this method is easier to perform compared with LAD ligation in neonatal mice and its success rate is higher. Given the small size of neonatal mice, cardiac surgery must be performed under a surgical microscope and the entire process should not take more than 10 min, as the duration of hypothermia is an important factor for survival rates.

## Materials

### Biological materials

- **Mice:** we have established the surgery in a mixed ICR/CD1 (Charles River Laboratory) and C57BL/6 background. However, this method can be applied to any mouse strain.
  **Critical**
  If the line used is purely C57BL/6, we recommend using ICR/CD1 females as foster mothers, because of their good nursing behavior.
  **Caution**
  All animal procedures must be conducted according to relevant institutional and governmental rules and regulations. All animal procedures were conducted in accordance with the guidelines from Directive 2010/63/EU of the European Parliament on the protection of animals used for scientific purposes. The experiments were approved by the local state authorities (84-02.04. 2014.A161 and 81-02.04. 2020.A463 Landesamt für Natur, Umwelt und Verbraucherschutz Nordrhein-Westfalen, Germany).

### Reagents

- Buprenorphine
  **Caution**
  Buprenorphine is a psychotropic substance, falling under governmental control internationally. Obtention, usage and disposure of such substances must comply with the relevant laws and regulations in local jurisdiction. Its usage must be carefully documented and reported to the authorities.
- Isoflurane
  **Caution**
  Isoflurane is an anesthetic gas and may be subject to authorization in different regions. Anesthetic devices containing isoflurane must be equipped with a scavenging system for the removal of the excess/waste isoflurane. Anesthetic devices should be operated in a well-ventilated room. Store the bottle of isoflurane in a locked drawer at room temperature (18–25 °C)
- Hair removal cream (Veet, cat. no. 07768282)
- Ultrasound gel, Aquasonic (Parker, cat. no. BT-025-0039N)
- 1× phosphate-buffered saline (PBS)
- 0.5 M potassium chloride (KCl)
- 4% paraformaldehyde (PFA)
- 30% sucrose–PBS solution
- Tissue-Tek OCT compound (Sakura, cat. no. 4583)
- Bouin’s Solution (Sigma, cat. no. HT101128)
- 0.1% Fast Green (Sigma, cat. no. F7258)
- 1% acetic acid
- 0.1% Direct Red 80 (Sigma, cat. no. 365548)
- Isopropanol
- Xylene
- Entellan (Merck, cat. no. HX90554761)
- 0.2% Triton X-100
- 5% normal donkey serum
- Primary antibodies of choice (recommended: ACTN2: 1:200, Sigma A7811; AURKB: 1:200, BD Biosciences 611082; cCasp3: 1:50, Cell Signaling 9441L; CD45: 1:1,000, Millipore CBL1326; GFP: 1:400, Abcam ab6662; Ki67: 1:200, Thermo Fisher RM-9106; P21: 1:100, Abcam ab109199; PECAM1: 1:500, BD Pharmingen 550274; TNNI3: 1:200, Abcam ab56357)
- Secondary antibodies of choice conjugated to Cy2, Alexa Fluor 488, Cy3 or Alexa Fluor 647, respectively (Jackson ImmunoResearch)
- 4,6-Diamidino-2-phenylindole dihydrochloride (DAPI; Invitrogen, cat. no. R37606)
- Aqua-Poly/Mount (Polysciences, cat. no. 18606)
- Fluorescein-labeled wheat germ agglutinin (WGA; Vector Labs, cat. no. FL1021)

### Equipment

#### Critical

**Fig. 1:**
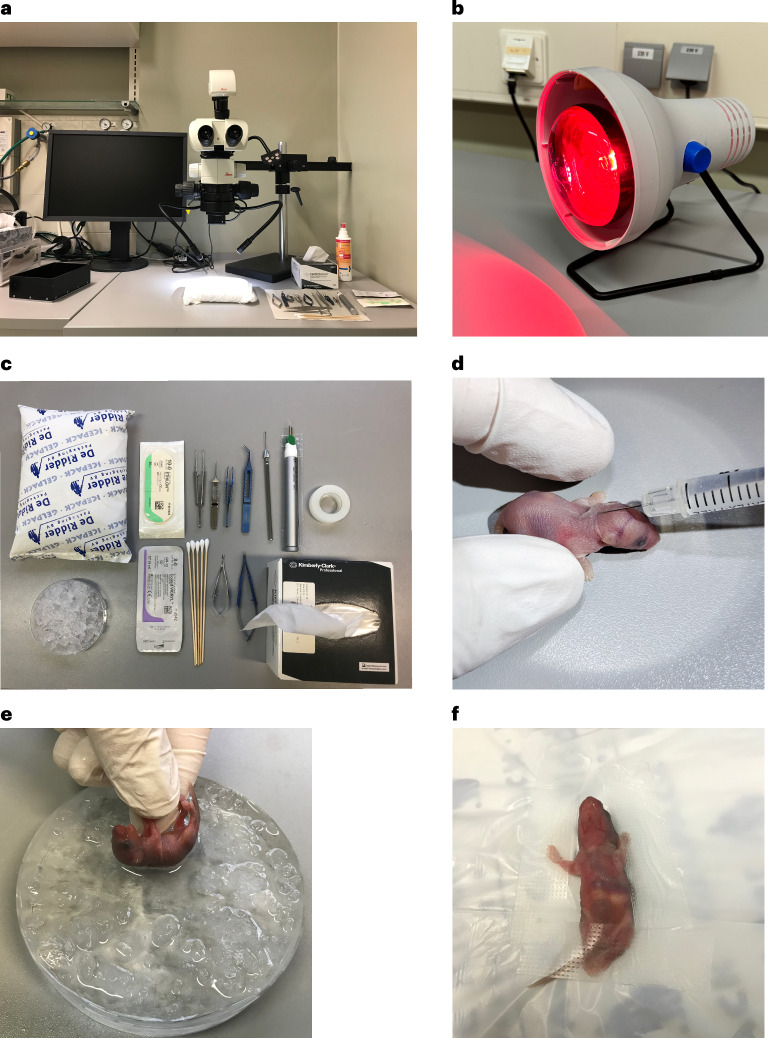
Surgery setup and preparation. **a**, Surgery setup including the surgical microscope, tools and equipment. **b**, A heating lamp for postsurgery recovery. **c**, Surgery tools and materials required for cauterization surgery. **d**, Subcutaneous injection of buprenorphine in P1 mice. **e**, Anesthesia using an ice-water bath. **f**, Fixed pup on an ice pad for the surgery. Appropriate institutional regulatory board permission was obtained for these experiments.
  **Critical step**
  Buprenorphine must be also injected 6 h after surgery and once daily for 3 days after surgery.
  **Caution**
  The analgesia regime has to be adapted on the basis of the approved protocol by the corresponding authorities.

Operation setting and equipment required for the procedure are shown in Fig. 1a–c.

- Frozen ice pack (containing sodium polyacrylate) to use as an ‘operating table’
- Q-tips (autoclaved)
- Kimberly-Clark Professional Kimtech Science Precision Wipes Tissue Wipers
- Glass Petri dish filled with ice water
- Surgical tape, 1.25 cm × 9.2 m, Leukofix (Leukoplast, cat. no. 02136-00)
- Heating lamp (Sanitas, cat. no. 614.21)
- Heating box with controlled temperature (self-made)
- M80 Leica microscope including vertical illumination (Leica, cat. no. LED3000 NVI)
- Ultrasound device, Vevo 3100 (VisualSonics) with a 50 MHz transducer (MX700) and 3D motor (optional)
- Heating device for warming ultrasound gels (Thermasonic Gel Warmer, cat. no. 82-03)
- Moria nickel plated pin holder, jaw opening diameter: 0–1 mm, alloy/material: nickel, length: 12 cm (Fine Science Tools (FST), cat. no. 26016-12)
- Insert pins, rod diameter: 0.6 mm, alloy/material: stainless steel, length: 5 cm, tip shape: triangle (FST, cat. no. 26007 -04)
- 10–0 suture Ethilon (Ethicon, cat. no. EH7467G)
- 8–0 coated Vicryl suture (Ethicon, cat. no. V542G)
- Cryostat (Leica CM3050)

### Surgical tools

- McPherson forceps, length: 11.5 cm, tips: 45 °C angle, 6 mm tying platform, titanium (World Precision Instruments (WPI), cat. no 555009FT)
- McPherson Forceps, length: 8.5 cm, tips: angled, 4.5 mm tying platforms, titanium (WPI, cat. no. 555005FT)
- Semken forceps with serrations, tips: serrated, tip shape: curved, tip width: 1.3 mm, Tip dimensions: 1.3 × 1 mm, alloy/material: stainless steel, length: 13 cm (FST, cat. no. 11009-13)
- Mirror finish suture tying forceps with 1 × 2 teeth, tip shape: straight, tips: 1 × 2 teeth, tip width: 0.3 mm, tip dimensions: 0.4 × 0.3 mm, platform length: 6 mm, alloy/material: stainless steel, length: 9 cm (FST, cat. no. 11090-09)
- Vannas spring scissors – microserrated, tips: sharp, tip diameter: 0.1 mm, alloy/material: stainless steel, serrated, tip shape: straight, cutting edge: 4 mm, length: 8.5 cm (FST, cat. no. 15070-08)
- Curved needle holder, titanium, 11 cm long (4.3 in.), curved, 6 mm delicate jaws, without lock (WPI, cat. no. 555408NT)

### Cauterization tools

- Small vessel cauterizer kit, material: stainless steel autoclave safe, handle length: 13.5 cm, handle weight: 100 g (FST, cat. no. 18000-00)
- Replacement tips for small vessel cauterizer alloy/material: platinum-iridium, tip shape: straight knife, tip diameter: 0.3 mm (FST, cat. no. 18000-03)

## Procedure

### Critical

When undertaking sham surgery, carry out all the steps described below including removal of the pericardium, except cauterization of the LCA (Step 14).

### Surgery preparation (day 1)

Timing 5 min per mouse pup

1. Before surgery, disinfect the working area, surgical tools and consumables either by autoclave or 70% ethanol.
2. Relocate half of the pups at a time from the mother into a box, which contains the bedding from the mother’s cage and is located on a heating plate set to 37 °C. Keep the mother’s cage away from the pups.
  **Critical step**
  Do not take the pups one by one from the mother’s cage, as this will induce stress and increase cannibalization rate.
3. Weigh the pups and inject buprenorphine (0.05 µg per gram body weight) subcutaneously (Fig. 1d), 30 min before the surgery.

### Anesthesia and surgery (day 1)

Timing 10 min per mouse pup

#### Critical

Refer to Supplementary Video 1 for visualization of surgery procedures.

4. After 30 min of buprenorphine injection, induce hypothermia by placing the pup in an ice-water bath for 2–3 min, while holding the legs (Fig. 1e).
  **Critical step**
  Do not place the mouse in direct contact with ice as the skin is very thin and fragile and this can result in frostbite.
  **Critical step**
  Stop hypothermia as soon as there is no paw reflex upon a gentle paw pinch with forceps. Prolonged hypothermia influences mortality and recovery rate.
5. Remove the pup from the ice-water bath, and gently dry the pup skin with a paper towel.
6. Place the pup in a supine position on an ice pack covered with a thick plastic cover to prevent the pup from coming into direct contact with the cold surface (Fig. 1f).
  **Critical step**
  Do not use the ice pack immediately after taking it out of the −20 °C freezer. Prolonged exposure (10 min) to low temperature (below −10 °C) during surgical procedure can kill the pups. To ensure proper temperature of the operating table, make sure the surface area of the ice pack is not very cold and that the ice pack is slightly melted. Take the ice pack out at least 30 min before and/or keep it under running hot water to acclimate to the desired temperature (0–4 °C) and cover it with a thick disinfected plastic sheet. If necessary, test the surface temperature with an infrared thermometer.
  **Critical step**
  The ice pack should not be too warm (above 8 °C) as this prevents full anesthesia and the pup could wake up during the procedure.
  **Critical step**
  As the ice pack gets warmer after a few surgical procedures, it has to be exchanged with a cooler one. Therefore, make sure to prepare backup ice packs before operating.
7. Fix the right arm and the legs flat on the ice pack with tape strips (Fig. 1f).
8. Fix the left arm so that it is a little bit lifted and not flat on the ice (Fig. 1f); this will create more space and flexibility for the surgery.
9. Disinfect the skin at the location of the surgery, and position the ice pack and the pup under the microscope with the head upside.
10. Under the microscope, first create a skin incision by lifting and cutting the skin starting from approximately 1 mm to the left of the xiphoid process and cut in parallel to the sternum until the opening reaches the manubrium (Fig. 2a); then hold the muscle underneath the skin and cut it open (Fig. 2b,c).

**Fig. 2:**
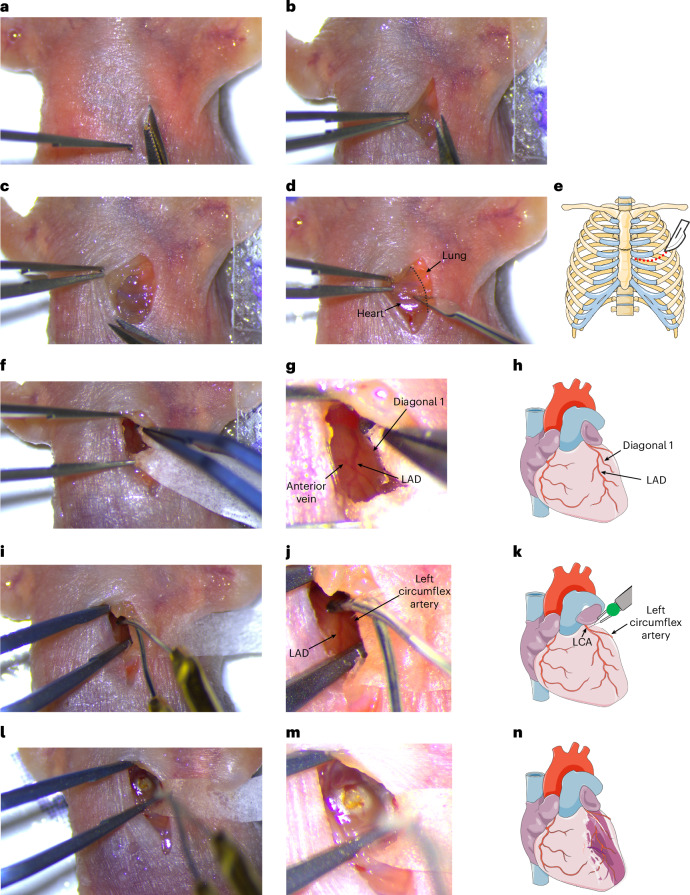
Schematic and microscopic views of the surgery procedures. **a**, Generation of a skin incision by cutting the skin. **b**, Holding the underneath muscle. **c**, Cutting the muscle underneath the skin. **d**, Lateral thoracotomy at the fourth intercostal space. **e**, Schematic image showing the anatomical location of the lateral thoracotomy by red dashed line. **f**, Removing the pericardium. **g**, Microscopic image showing the location of the LAD. **h**, Schematic image showing the anatomical location of the LAD. **i**, Microscopic image showing cauterization of the root of the LCA. **j**, Higher magnification of microscopic image of the heart showing cauterization of the root of the LCA. **k**, Schematic image showing the anatomical location of the LCA and cauterization site. **l**, Microscopic image showing the cauterized area in the heart. **m**, Higher magnification of microscopic image of the heart after cauterization of the root of the LCA. **n**, Schematic image showing the ischemic area in the heart after cauterization of the root of the LCA. Appropriate institutional regulatory board permission was obtained for these experiments. The schematic images were obtained from Servier Medical Art with modifications (https://smart.servier.com/).
  **Critical step**
  The size of the opening depends on how skilled the surgeon is to find and visualize the LCA upon opening. Therefore, practice is required to make a smaller incision.
11. Perform lateral thoracotomy at the fourth intercostal space (Fig. 2d,e).
12. Remove the pericardium to expose the heart surface. Insert a tissue stipe to absorb the pericardial effusion when necessary (Fig. 2f).
Troubleshooting
13. Lift the left atrium gently with blunt ends forceps to visualize the root of the LCA (Fig. 2g,h).
  **Critical step**
  Lift the atrium gently and only with blunt-ends forceps because the neonatal atria are very thin and easy to rupture. The apex of the atrium covering the cauterization site is often slightly damaged, without adverse effect on the regenerative process. This is either due to its chafing against the hardened surface of the cauterization site and/or the uplifting with the forceps despite careful manipulation.
14. Perform the cauterization of the LCA at approximately 1–2 mm distal to the left coronary orifice using the vessel cauterizer (Fig. 2i–k); confirm the cauterization by observing the paling of the anterior part of the LV (Fig. 2l–n).
  **Critical step**
  Be careful not to touch anywhere else with the cauterizer as it can burn and damage other tissues or areas, leading to bleeding or death.
15. Close the chest by suturing the muscles above the ribs using an 8–0 absorbable suture (Fig. 3a–c).

**Fig. 3:**
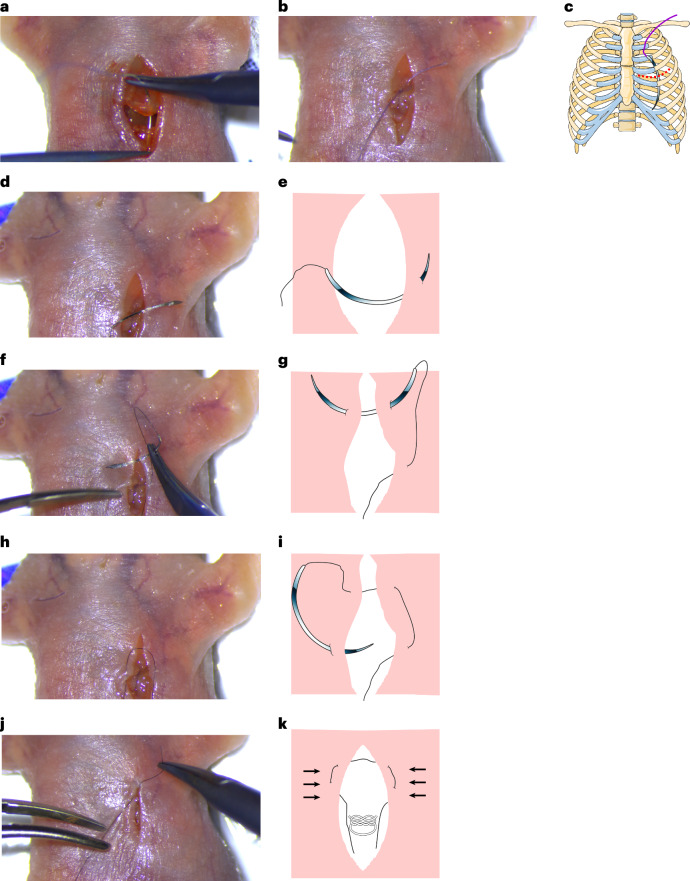
Closing the ribs and the skin. **a**–**c**, Suturing the ribs using an absorbable suture. Suturing the ribs by entering the needle from the upper to the lower side of the incision (**a**). Making a knot to close the ribs (**b**). Schematic image showing the location of the suture and the incision (red dashed line; **c**). **d**–**k**, Step-by-step procedures to close the skin in a way to conceal the suture and the knot inside the skin. Suturing the skin by inserting the needle from the inner side of the middle right part of the skin (**d**). Schematic image showing the location of suture to enter the skin (**e**). Inserting the needle from the outside of the upper right part of the skin incision to the inner side of the upper left part of the skin (**f**). Schematic image showing the position of the needle in the skin (**g**). Inserting the needle from the outside of the lower left part of the skin incision (**h**). Schematic image showing the location of the needle to enter the skin in this step (**i**). Making a surgeon’s knot inside of the skin incision (**j**). Schematic image showing the location of the knot (**k**). Appropriate institutional regulatory board permission was obtained for these experiments. The schematic images were obtained from Servier Medical Art with modifications (https://smart.servier.com/).
Troubleshooting
16. Suture the skin with a 10–0 nonabsorbable suture. To hide the knot, start by inserting the needle from the inner side of the middle right part of the skin incision (Fig. 3d,e). After pulling out the needle from the outside of the skin, insert the needle from the outside of the upper right part of the skin incision, and continue to the inner side of the upper left part of the skin incision (Fig. 3f,g). After pulling out the needle, insert the needle again from the outside of the lower left part of the skin incision (Fig. 3h,i). To finish, make a surgeon’s knot inside of the skin incision (Fig. 3j,k). In this case, the knot and most part of the sutures will bury underneath the skin. Repeat the same method for suturing the lower part of the skin incision, and hide the knot inside the skin (Fig. 4a–d).

**Fig. 4:**
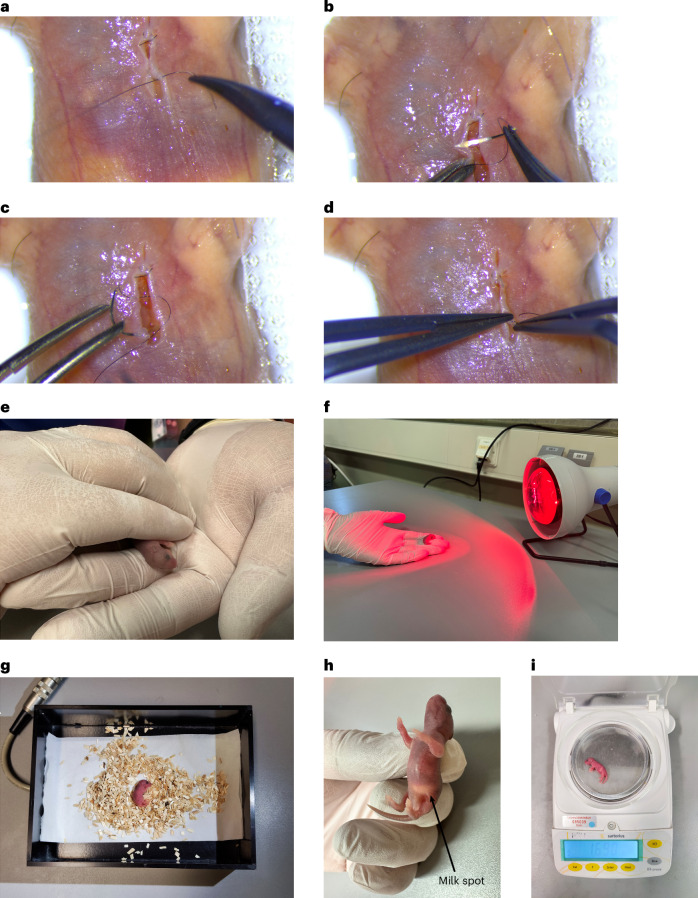
Closing the skin and postoperative recovery and follow-up procedures. **a**–**d**, Complete skin closure by applying the second suture. Suturing the lower part of the skin by inserting the needle from the inner side of the middle right part of the skin incision (**a**). Inserting the needle from the outside of the upper right part of the skin incision to the inner side of the upper left part of the skin incision (**b**). Inserting the needle from the outside of the lower left part of the skin incision (**c**). Making a knot inside of the skin incision (**d**). **e**–**g**, Postsurgical recovery process including warming the pup first in hands (**e**), then shortly in front of a heating lamp (**f**) and then in a warm box set to 37 °C (**g**)**. h**,**i**, Postsurgery monitoring by visualizing the presence of a milk spot (**h**) and controlling the body weight (**i**). Appropriate institutional regulatory board permission was obtained for these experiments.
  **Critical step**
  Our suture method for the skin was modified from the horizontal mattress suture^29^ and subcuticular closure suture^30^, so that the knot falls inside of the skin and the suture is mostly invisible from the surface. In our experience, this method avoids the suture to be bitten away by the mother during postsurgical nursing, thus reducing mortality and cannibalization rate.
17. Remove the pup by gently removing the tapes and warm the pup in the hands (Fig. 4e); then shortly keep the pup in front of a heating lamp to recover (Fig. 4f), which is observed by spontaneous breathing.
  **Critical step**
  The skin is very fragile; be careful not to rip apart the skin when removing the tapes.
  **Critical step**
  When warming the pup with the lamp, do not keep it too close to the lamp as this can cause burn injuries and increase mortality.
18. Then, place the operated pup in a heating box set to 37 °C containing bedding from the mother cage for recovery (Fig. 4g).
19. When the surgery is done for half of the litter and they are fully recovered and active, return the pups to the mother’s cage by replacing them with the other half of the litter and mixing them with the bedding.

### Follow-up and monitoring of the mice postsurgery (days 1–7)

Timing 5 min per mouse pup

20. Check the pups once per day for their ability to drink milk, weight gain, body temperature, suture integrity and acceptance by the mother. The milk spot should be clearly visible (Fig. 4h), and the pups should gain weight regularly, which can be checked by measuring their weight (Fig. 4i). Buprenorphine should be injected 6 h postsurgery and once per day for 3 days after surgery.
  **Critical step**
  Score the health/suffering of the pups on the basis of the criteria mentioned in the approved animal protocol to evaluate their health and conditions postsurgery, and kill them on time if their development is impaired or they are not in a good condition, as mentioned and approved in the score sheet of your approved animal protocol.

### Confirmation of successful surgery and follow-up cardiac function using echocardiography (days 1–120)

Timing 10 min per mouse pup

#### Critical

We recommend confirming the success of the surgery one day later by performing an echocardiography (Vevo 3100) to visualize the reduced movement of the LV free wall and consequently reduced EF. In addition, it is recommended to repeat the echocardiography at more time points after surgery to follow up the regeneration or lack or regeneration (Fig. 5a).

#### Critical

Prewarm the ultrasound gel (Fig. 5b) and the table before starting the procedure.

21. One day after surgery, place the pup in a supine position on the echocardiography heating table set to 37 °C (Fig. 5c).

**Fig. 5:**
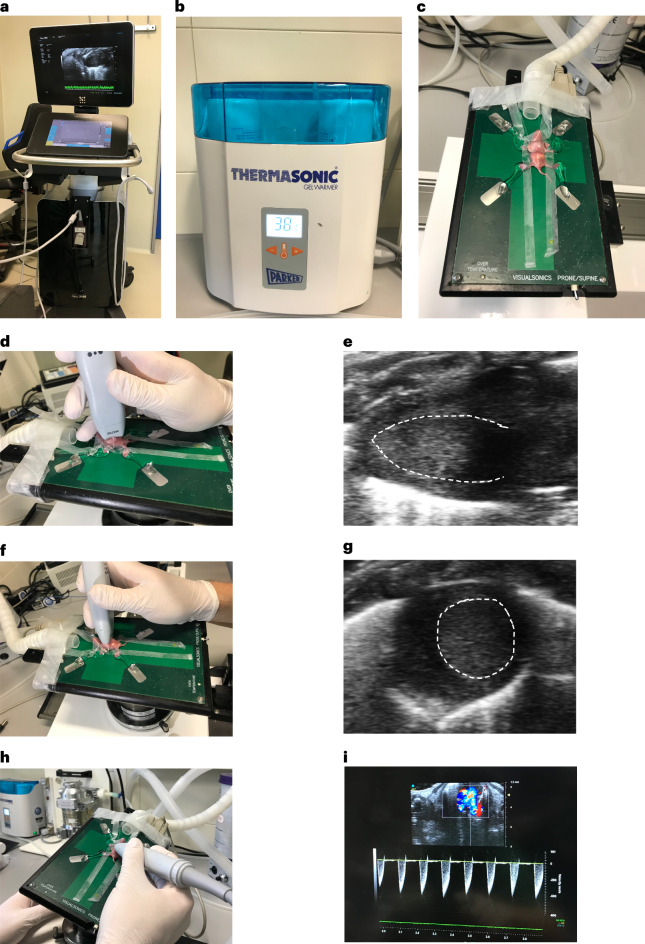
Echocardiography equipment and procedure. **a**, Echocardiography device (Vevo 3100, VisualSonics). **b**, Heating box for warming ultrasound gels. **c**, Fixed pup on the echocardiography table. **d**,**e**, Holding position of the transducer on the pup (**d**) for visualizing the long-axis view of the heart (**e**). **f**,**g**, Holding position of the transducer on the pup (**f**) for visualizing the short-axis view of the heart (**g**). **h**,**i**, Holding position of the transducer on the pup (**h**) for visualizing and measuring the flow velocity of the pulmonary artery (**i**). Appropriate institutional regulatory board permission was obtained for these experiments.
22. Fix the head and legs of the mouse with long strips of tape (Fig. 5c).
  **Critical step**
  While P1–P7 mice can be fixed on the heating plate with adhesive taps on their palms and chins without anesthesia, older mice need to be put under anesthesia (2–3% isoflurane with oxygen flow at 1 l min^−1^) and fixed with adhesive taps.
23. Apply a thick layer of prewarmed ultrasound transmission gel on the chest of the mouse (Fig. 5c).
  **Critical step**
  Keep the pups warm and use prewarmed ultrasound gel to avoid a decrease in body temperature, which could lead to a decrease in heart rate and influence cardiac functional parameters.
  **Critical step**
  Depending on the age of the animal and the scanning depth of the transducer, different amounts of gel must be applied.
24. Owing to the small size of the pup, the paws do not reach the electrocardiogram recording electrodes on the table; thus, to record an electrocardiogram, ultrasound transmission gel has to be drawn from the paws to the electrodes (Fig. 5c).
25. Use a Mx700 transducer (from 1 to 14 dps) of VisualSonics Vevo 3100 ultrasound device, which has a bandwidth of 21–70 MHz and scanning depth of 10 mm, to visualize the heart in the long-axis view in B mode (Fig. 5d,e) and short-axis mode (Fig. 5f,g). For later time points (from 21 to 120 dps), a Mx550 transducer can be used. Record a few long-axis views and short-axis views of the heart for further analysis using offline Vevo LAB software.
  **Critical step**
  Removal of body hair on the chest area is required for echocardiography of pups from 14 days of age.
  **Critical step**
  Reduced EF and movement of LV free wall should be measured in the long axis view in B mode, as this allows assessment of the entire LV regardless of the ischemic area or border zone.
  **Critical step**
  To standardize imaging and ensure better reproducibility of the data, capture the long- and short-axis views of the hearts always at the middle part of the heart, by visualizing the hallmark of papillary muscle or aortic valve in the short-axis or the long-axis view, respectively.
  **Critical step**
  LV function and parameters such as EF, FS, LVEDA and CO are recommended to be determined on the basis of long-axis view images; however, LV wall thickness can be measured in the short-axis view using the integrated measurement tools of the Vevo Lab 3.2.6 software.
  **Critical step**
  To assess the cardiac parameters, systolic and diastolic areas must be measured within the same heart cycle. To prevent false interpretation, it is recommended to measure and average the parameters three times using different heart cycles.
  **Critical step**
  RVEDA and RVFAC can be measured in the short-axis view and can be given as percentages using 100 × (RVEDA − RVESA)/RVEDA.
  **Critical step**
  Pulmonary arterial pressure can be measured using pulsed-wave Doppler of the main pulmonary artery (Fig. 5h). The correct location of the sampling gate has to be assured in color Doppler imaging (Fig. 5i). Pulmonary artery acceleration time can be measured as the time from the onset of systolic pulmonary blood flow to the peak flow velocity; pulmonary artery ejection time can be measured as the total time of systolic pulmonary blood flow^31,32^.
26. After scanning, remove the gel and the tapes gently without damaging the skin.
27. After finishing the echocardiography, return the pups to the mother’s cage and download the files from the machine.

### End of study: organ removal for histological and molecular/cellular analyses

Timing 10 min

28. Kill the mice according to the approved institutional methods, by decapitation or cervical dislocation, and collect the heart.
  **Critical step**
  Open the chest and the ribs carefully because the heart might have adhered postoperatively to the surrounding tissues such as ribs or lungs.
29. Wash the isolated hearts in cold PBS, 1×. Let it pump the blood out and gently squeeze the ventricles. Then, place the heart into 0.5 M KCl to relax the cardiac muscle before fixation to achieve a diastolic morphology for histology.
30. Measure heart weight to tibia length and/or heart weight to body weight ratios.

### Histology and immunofluorescence staining

Timing 2 days

31. Fix the hearts for 1 h at 4 °C in 4% PFA.
32. Wash the heart with cold PBS (4 °C) for 5 min, three times.
33. Equilibrate the heart in 30% sucrose–PBS solution overnight at 4 °C.
34. For cryo-sectioning, embed the hearts in Tissue-Tek OCT compound and cut into 7–10-μm-thick sections with a cryostat.
35. For assessing the initial scar size, perform immunostaining using TNNI3 staining and/or TUNEL assay as the fibrotic scar has not developed yet. The scar can be visualized in the LV by the lack of TNNI3 staining (Fig. 6a,b) and/or positive TUNEL cells.

**Fig. 6:**
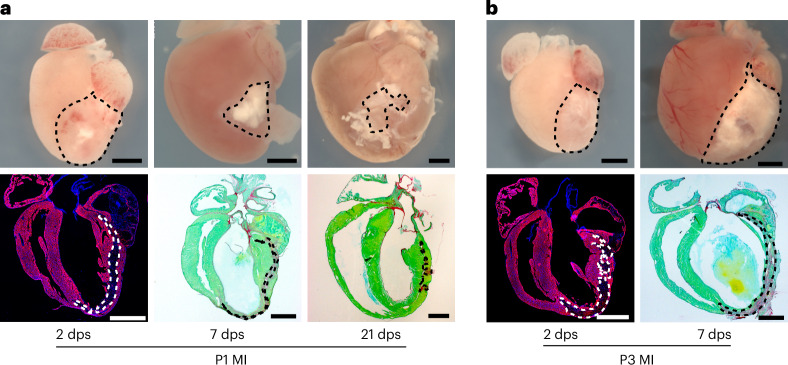
Overview and histological images of the heart after P1 and P3 cauterization. **a**, P1 MI hearts (top) and heart sections (bottom) at 2, 7 and 21 dps. Visualization of the initial infarct area (marked with dashed lines) at 2 dps with TNNI3 and DAPI (bottom left picture) or the scarred area after 7 dps and 21 dps with Sirius Red and Fast Green co-stainings (bottom middle and right pictures). Scale bars, 1 mm. **b**, P3 MI hearts (top) and heart sections (bottom). Visualization of the initial infarct area (marked with dashed lines) with TNNI3 and DAPI (bottom left picture) or scar area with Sirius Red and Fast Green co-stainings (bottom right picture). Scale bars, 1 mm.
  **Critical step**
  For quantitative measurements, analyze a minimum of seven sections across the entire heart at approximately equal intervals, as described before^33^. The size of the lesion can be calculated as percentages by normalizing the lesion area to the total LV area from all sections and multiplying the number by 100.
36. To determine the scar size after 7 dps and later, use Sirius Red staining with Fast Green counterstaining (Fig. 6a,b). Rehydrate the heart sections in PBS and fix in Bouin’s Solution at 55 °C for 1 h. After washing the heart sections with water for 10 min three times, incubate them in 0.1% Fast Green for 10 min, then fix in 1% acetic acid for 2 min, and incubate in 0.1% Direct Red 80 for 30 min. After washing with water for 5 min, three times, dehydrate the slides by passing through three changes of isopropanol, then three times in xylene and mount with Entellan.
37. To assess processes such as proliferation, apoptosis and angiogenesis using immunostaining, fix heart sections for 10 min in 4% PFA. After washing with PBS for 5 min, three times, block nonspecific antigens for 30 min using PBS containing 0.2% Triton X-100 and 5% normal donkey serum. Dilute the primary antibodies of choice (recommended: ACTN2: 1:200; AURKB: 1:200; cCasp3: 1:50; CD45: 1:1,000; GFP: 1:400; Ki67: 1:200; P21: 1:100; PECAM1: 1:500; TNNI3: 1:200) in PBS containing 0.2% Triton X-100 and 2.5% normal donkey serum. Then, apply the antibodies to the sections followed by an overnight incubation at 4 °C. After washing with PBS for 5 min, three times, apply diluted (in PBS) secondary antibodies conjugated to Cy2, Alexa Fluor 488, Cy3 or Alexa Fluor 647, respectively (all 1:400) and 0.5 µg ml^−1^ DAPI. Incubate for 30–60 min at room temperature. Wash the sections with PBS for 5 min, three times, and mount the slides with Aqua-Poly/Mount.
38. For assessing CM hypertrophy after MI, stain the sections with WGA and measure CM cross-sectional area. This staining can be done similarly to the immunostainings, except for an additional 1 h incubation in fluorescein-labeled WGA (1:1,000 dilution in PBS) before mounting.
  **Critical step**
  It is recommended to quantify only CM with nuclei and symmetrical cross-sectional shapes in the images.

## Troubleshooting

Troubleshooting advice can be found in Table 1.

**Table 1:** Troubleshooting table

Step	Problem	Possible reason	Solution
4	Mortality	Hypothermia duration was too long	Check the pups regularly for paw reflex, and stop hypothermia as soon as no reflex is visible
6	Mortality	The pup freezes	The ice pad was too cold. Prewarm the ice pad
	The pups wake up	Insufficient hypothermia	Duration of hypothermia before transferring the pup on ice pad should be enough, and ice pad also should be cold enough. Change ice pad when necessary
12	Bleeding inside the chest	The heart has been damaged when removing the pericardium; and/or the lung has been damaged	The pericardium is very thin. Try not to touch the heart while removing the pericardium, and do not use sharp forceps. Make sure not to puncture or slash other tissues inside the chest wall
13	Excessive bleeding inside the chest	The atrium or the heart has been damaged	Do not hold the atrium. Just lift it up by going underneath using blunt forceps
15	Bleeding outside the chest	The ribs have been damaged	Make sure to insert the suture into the muscle, not the ribs
17	Mortality	The procedure and hypothermia were very long, or bleeding occurred during surgery	Practice the surgery in advance, focusing on critical steps to speed up the surgery and prevent bleeding and damaging other organs than the heart
20	Cannibalization	Young, stressed mother. Uneven, messy skin closure	Use ICR/CD1 mothers. Prevent too much stress, and suture the skin as tidy as possible

## Timing

Per mouse pup:

Steps 1–3, preparation for surgery: 5 min

Steps 4–5, anesthesia: 2–3 min

Steps 6–17, surgery: 6–10 min

Steps 18–19, recovery and immediate follow-up: 5–10 min

Step 20, follow-up, postsurgery control and analgesia administration: 5 min

Steps 21–27, echocardiography: 10 min

Steps 28–30: heart isolation: 10 min

Steps 31–38: histology and immunofluorescence staining: 2 days

## Anticipated results

In our recently published study, the cauterization method was applied to both P1 and P3 mice, resulting in a similar initial injury size at 2 dps corresponding to 40% of the LV, located in the myocardium layer of the LV^7^ (identified using immunostaining against TNNI3, Step 35). In P1 mice, the injury size significantly decreased to approximately 20% and further to 6% of the LV at 7 and 21 dps, respectively. However, in P3 mice the scar size increased in size to almost 70% of the LV and became transmural (identified using Sirius Red staining, Step 36). The surgery did not influence natural growth of the pups in P1, and mortality rate was 10%. However, mortality increased gradually in P3 mice, which displayed signs of impaired growth by 7 dps. Consequently, we opted to kill the remaining P3 operated mice at P7 to adhere to animal welfare guidelines.

Similarly, postsurgical monitoring and evaluation of cardiac function by echocardiography revealed comparable reduction in cardiac pump function (EF of 20%) in both P1 and P3 operated mice at 1 dps. It is highly recommended to conduct echocardiographic assessment of heart function at 1 dps, as it provides functional evidence of successful surgery and similarities among the pups/genotypes. Although cardiac pump function gradually improved in P1 operated mice and reached almost 60% by 7 dps, it remained low (20%) in P3 operated mice at 7 dps. We assessed RV function by measuring RVFAC (a two-dimensional measure of right ventricular global systolic function) in the short-axis view of the heart at 1 and 7 dps and could identify preserved RV function in P1 operated mice, despite signs of pulmonary hypertension indicated by reduced pulmonary artery acceleration time/pulmonary artery ejection time. RV function, however, diminished in P3 operated mice immediately after injury and remained low at 7 dps^7^.

On a cellular and molecular level, we identified enhanced CM proliferation and binucleation (CMs with two nuclei, indicating mitosis in the absence of cytokinesis) rates in the RV as well as the LV of P1, but not P3 mice. At 7 dps, capillary density in the LV of P1 operated mice returned to the levels seen in control uninjured hearts^7^, after being reduced following ischemic injury^22^. This phenomenon was interestingly associated with enhanced angiogenesis of the RV. The injury in P3 mice, however, triggered widespread apoptosis and CM hypertrophy. In our recent publication, we have provided sequencing data obtained from the RV, LV and septum of P1 and P3 operated mice at 1 dps^7^. Given the good health of P1 mice after operation, owing to their ability to regenerate the heart, we were able to keep them until 120 dps. Examination of the regenerated P1 heart at 120 dps unveiled relatively minor structural and morphological changes^7^; in particular, a very small scar (2.92 ± 0.10% of total LV area) was found.

Our cauterization protocol provides a useful model to study cardiac regeneration in P1 mice and lack thereof in P3 mice. This surgical method has effectively confirmed that proper cardiac regeneration is abolished by P3. In addition, this model can be used to study the adaptive response of the RV to secondary pulmonary hypertension. Deciphering the cellular and molecular mechanisms underlying LV and RV plasticity, adaptation and regeneration after MI could reveal novel as well as improve existing therapeutic strategies in the future.

## Supplementary information

Supplementary Video 1 Video demonstrating surgical procedures involved in cauterization of the root of the LCA in P1 mouse.

## Data Availability

All RNA-sequencing data sets, as reported in the related manuscript^7^, are deposited in the Short Read Archive at the NCBI under BioProject ID PRJNA941198. Data associated with the quantifications of scar, echocardiography data and further relevant quantifications such as CM proliferation and angiogenesis are all available and published as the supporting data values, Excel file or supplementary table under 10.1172/jci.insight.176281 (ref. ^7^).
